# Perceived sounds and their reported level of disturbance in intensive care units: A multinational survey among healthcare professionals

**DOI:** 10.1371/journal.pone.0279603

**Published:** 2022-12-30

**Authors:** Nicole Ruettgers, Aileen C. Naef, Marilyne Rossier, Samuel E. J. Knobel, Marie-Madlen Jeitziner, Martin Grosse Holtforth, Bjoern Zante, Joerg C. Schefold, Tobias Nef, Stephan M. Gerber

**Affiliations:** 1 Gerontechnology and Rehabilitation Group, ARTORG Center for Biomedical Engineering Research, University of Bern, Bern, Switzerland; 2 Department of Intensive Care Medicine, Inselspital, Bern University Hospital, University of Bern, Bern, Switzerland; 3 Institute of Nursing Science (INS), Department of Public Health (DPH), Faculty of Medicine, University of Basel, Basel, Switzerland; 4 Psychosomatic Medicine, Department of Neurology, Bern University Hospital, University of Bern, Bern, Switzerland; 5 Department of Clinical Psychology and Psychotherapy, University of Bern, Bern, Switzerland; 6 Department of Neurology, Inselspital, Bern University Hospital, University of Bern, Bern, Switzerland; University of Verona, ITALY

## Abstract

**Purpose:**

The noise levels in intensive care units have been repeatedly reported to exceed the recommended guidelines and yield negative health outcomes among healthcare professionals. However, it is unclear which sound sources within this environment are perceived as disturbing. Therefore, this study aimed to evaluate how healthcare professionals in Germany, Switzerland, and Austria perceive the sound levels and the associated sound sources within their work environment and explore sound reduction strategies.

**Material and methods:**

An online survey was conducted among 350 healthcare professionals working in intensive care units. The survey consisted of items on demographic and hospital data and questions about the perception of the sound levels [1 (strongly disagree) to 5 (strongly agree)], disturbance from sound sources [1 (not disturbing at all) to 5 (very disturbing)], and implementation potential, feasibility, and motivation to reduce sound reduction measures [1 (not high at all) to 5 (very high)].

**Results:**

Approximately 69.3% of the healthcare professionals perceived the sound levels in the ICUs as too high. Short-lasting human sounds (e.g. moans or laughs) [m*ean (M)* ± *standard deviation (SD)* = 3.30 ± 0.81], devices and alarms (*M* ± *SD* = 2.67 ± 0.59), and short-lasting object sounds (*M* ± *SD* = 2.55 ± 0.68) were perceived as the most disturbing sounds. Reducing medical equipment alarms was considered to have greater implementation potential [*M* ± *SD* = 3.62 ± 0.92, t(334) = -7.30, *p* < 0.001], feasibility [*M* ± *SD* = 3.19 ± 0.93, t(334) = -11.02, *p* < 0.001], and motivation [*M* ± *SD* = 3.85 ± 0.89, t(334) = -10.10, *p* < 0.001] for reducing the sound levels.

**Conclusion:**

This study showed that healthcare professionals perceive short-lasting human sounds as most disturbing and rated reducing medical equipment alarms as the best approach to reduce the sound levels in terms of potential, feasibility, and motivation for implementation.

## Introduction

The sound pressure levels, commonly referred to as simply the sound levels, in intensive care units (ICUs) far exceed the public health recommendations of 35–40 dBA [1–7]. Not only is this detrimental to patients exposed to noise while hospitalised, but it also affects healthcare professionals working in hospitals [8–17]. Among other effects, acute exposure to high noise levels has also been found to cause tachycardia, while chronic exposure has been linked to hypertension, heart disease, and stroke [18, 19]. However, it remains unclear how healthcare professionals working in this environment perceive their soundscape and what aspects they interpret as the most disturbing.

Many studies focus only on a few overarching sound sources, such as alarms, and fail to investigate the soundscape in more detail [20–24]. More specifically, most studies fail to address non-acoustical factors, which include the comprehensible content of sound (e.g. telephone conversations or discussions between colleagues) as well as the predictability, avoidability, controllability, task demands, and attitudes towards the sound source [15]. Instead, studies use the overall sound levels as a generalised explanation for noise pollution [3, 6, 15, 21, 25–29]. Accordingly, impulsive or sudden sounds are usually considered more disturbing than continuous sounds because individuals are required to expend more information-processing resources to understand them and are unable to develop effective coping strategies [17].

Based on this notion, simply identifying which sound sources produce the highest sound levels in ICUs is insufficient. How healthcare professionals working in ICUs perceive their soundscape and which sound sources they feel to be the most bothersome must first be evaluated to better understand how to improve their work environment. Therefore, this study aimed to explore how healthcare professionals in three German-speaking European countries perceive the sound levels in ICUs and their sources, as well as the attitudes towards sound reduction measures. It was hypothesised that short-lasting and sudden sounds are perceived as the most disturbing sounds, and that healthcare professionals prefer sound reduction measures that require the least behavioural adaptations related to their daily routine. Understanding these perceptions and attitudes is important for furthering research and creating policies that can improve the health and safety of healthcare professionals working in ICUs.

## Materials and methods

### Online survey distribution and ethics

Using a mix of purposive and random sampling, an online survey was sent to ICUs and intermediate care units (IMCs) in Germany, Switzerland, and Austria. Among hospitals with an ICU or IMC for adults, 200 were randomly selected from the 1250 units in Germany and 80 from the 130 units in Austria. In Switzerland, the survey was sent to all 84 ICUs and IMCs to achieve a similar sample size. A complete list of the ICUs and IMCs in each country of interest, and their contact information, is publicly available online. Using this information, the randomly selected units were contacted and asked to distribute the survey to their ICU staff. With the aim of increasing participation, and to ensure respondent anonymity, no questions requiring hospital identifying information was asked. No staff members were targeted in the distribution, with all individuals who received the survey able to participate, and even pass on the survey to colleagues. However, using this technique for distribution meant it was not possible to determine how many individuals the survey reached, and therefore, no response rate could be calculated.

Data collection lasted from June 2021 to February 2022, and the respondents completed the survey independently of the study team. The online survey was conducted using REDCap, an electronic data capture tool hosted in Switzerland, which enabled the construction and distribution of the survey and the collection and storage of the data [30, 31]. The respondents provided their informed consent online before answering the survey. This study was approved by our local ethics committee via a waiver, as no identifiable data were collected (KEK 2020–01294).

### Survey development and procedure

#### Hospital and demographic data

The first part of the survey included questions regarding hospital-related information, including the type of hospital, specialisation of unit, and type of unit (e.g. ICU or IMC). IMCs, also known as high-dependency or step-down units, are defined as those between ICUs and general units in terms of medical care intensity [32]. As the separation is not always clearly defined in all hospitals, the term ICU was used to describe both units in this study. Demographic information included sex, age, job, work experience, and employment.

#### Sound perception

The section on sound perception was divided into two parts: perception of the sound levels and disturbance caused by sound sources. The questions were based on items used in previous studies on the same topic [21, 29, 33]. The healthcare professionals were asked to rate their level of agreement on two statements (‘I perceive the sound level in my ward as too high’ and ‘I think the WHO guideline (35–40 dBA) can be met in my ward’) using a 5-point Likert scale to obtain interval-scaled datapoints with all points labelled from 1 (strongly disagree) to 5 (strongly agree). The detailed analyses regarding the perception of the sound levels were based on the first item. Additionally, the healthcare professionals were asked which work shift they perceived as the loudest.

Subsequently, the respondents rated the disturbance caused by various sound sources occurring during their work in the ICU. The sound sources queried were drawn from previous research [3, 4, 29, 34]. The list of 52 sound sources was organised into four sections. The first section contained different medical devices and their alarms, such as respirator or dialysis machines. The second section included all forms of communication, such as patient-related conversations, private conversations, laughs, or shouts. In the third section, the healthcare professionals were asked to rate the level of disturbance caused by sounds that occur daily, including shift change, patient entry, ward rounds, or standard care. When the respondents rated the sounds as partly, likely, or very disturbing, they were asked to elaborate on what exactly disturbed them about this sound source. This approach aimed at gathering more information on the quality of the disturbance, since the items did not strictly consist of a single sound. The fourth section contained all sounds generated by objects, such as phones, pagers, garbage bins, doors, drawers, and shoes. At the end of each section, the respondents had the opportunity to name additional sound sources they found disturbing.

The respondents rated each of the 52 sound sources on a 5-point Likert scale, with all scale points ranging from 1 (not disturbing at all) to 5 (very disturbing). For each sound, a ‘not present’ indication could also be made, as medical devices, work routines, objects, and architectural ward designs differ between hospitals. The respondents were advised that the survey was about disturbing sounds and not about how annoying they found certain tasks or processes in general. Answering all questions in the online survey was not mandatory; accordingly, not all healthcare professionals answered every item.

For the statistical analyses, the sound sources were categorised according to the origin (humans, objects, and medical devices and their alarms) and type (short-lasting or continuous) [7, 35, 36], resulting in the following five sound groups: devices and their alarms (e.g. vital sign monitor/alarms, respirator, or dialysis machine sounds), short-lasting object sounds (e.g. opening packages, squeaky shoes, or unmuted cell phones), short-lasting human sounds (e.g. calls over long distances, laughs, or complaining/moaning patients), continuous object sounds (e.g. cleaning wards, moving trolleys, or coffee machine sounds), and continuous human sounds (e.g. patient admission/takeover, daily nursing care, or visit from relatives). All sound sources, the order in which they were asked in the survey, and the assigned sound group are listed in S4 Table.

#### Sound reduction measures

The third part of the survey contained questions about sound reduction measures. Specifically, the respondents were asked whether enough effort was being made by employees and hospital managers to lower the sound levels in their ward. They were additionally asked whether their hospital had good equipment and infrastructure, such as insulated walls and windows or enclosed rooms for meetings, to reduce the sound levels [35]. Thereafter, the respondents were asked to rate six proposed sound reduction measures in relation to their potential, feasibility, and motivation for implementation. The proposed measures were as follows: 1) sensitise staff in general to keep conversations quieter; 2) alert employees if they are too loud; 3) include fewer people in ward rounds; 4) implement quiet hours; 5) modify the limit setting of alarms/adjust the monitoring intervals; and 6) acknowledge/turn off alarms more rapidly. For further analyses, measures one to four were analysed together as ‘changing staff behaviours and work procedures’, and measures five to six as ‘reducing medical equipment alarms’. Each measure was rated on a 5-point Likert scale, with all points labelled 1 (not high at all) to 5 (very high) and with potential, feasibility, and motivation each receiving their own rating.

### Statistical analysis

Initially, the hospital data, demographic data, perception of the sound levels, and disturbance from sound sources were tested for differences between countries. Next, descriptive analyses examining the distribution of the responses about the perception of the sound levels and sound reduction measures were conducted. For all statistical analyses, equality of variances was tested using Levene’s test. When equality of variances was met, a t-test and analysis of variance were used along with the post-hoc Bonferroni corrected pairwise t-test. Otherwise, the Kruskal–Wallis rank sum test along with the post-hoc Dunn–Bonferroni test was used for independent samples and the Friedman test along with the post-hoc Wilcoxon test for dependent samples. Data were analysed using SPSS (IBM SPSS version 28).

## Results

### Hospital and demographic data

A total of 350 healthcare professionals working in ICUs answered the questionnaire. As answering all questions was not mandatory, the total number of responses collected varied, with the percentages calculated according to the total data set available for each comparison. Of the participants, 37.4% (*n* = 131) were from Germany, 32.0% (*n* = 112) from Switzerland, and 30.6% (*n* = 107) from Austria. Approximately, 88.3% (*n* = 309) worked in a public or university hospital and 9.4% (*n* = 33) in a private hospital, and 2.3% (*n* = 8) declared another type of hospital or made no specification. Further, 74.6% (*n* = 261) worked in an ICU only and 3.7% (*n* = 13) in an IMC only, and 21.1% (*n* = 74) stated that there was no clear separation between the ICU and IMC in their hospital. No specification was made by 0.6% (*n* = 2). Approximately, 66.3% (*n* = 232) worked in a mixed unit, 14.9% (*n* = 52) in a surgical unit, and 9.1% (*n* = 32) in a medical unit. Meanwhile 6.6% (*n* = 23) worked in a specialised unit for diagnosis specific care, and 3.1% (*n* = 11) declared another type or made no specification. The most frequently represented units were ICUs with ≤ 20 beds (79.1%, *n* = 277), while the least frequently represented units were ICUs with > 20 beds (20.9%, *n* = 73). The participating units had predominantly multi-bed rooms (62.6%, *n* = 219), followed by a balanced number of single- and multi-bed rooms (19.7%, *n* = 69) and single-bed rooms (17.7%, *n* = 62). The hospital data are shown by country in S1 Table.

Of the respondents, 66.6% (*n* = 233) were women, and 32.9% (*n* = 115) were men; 0.5% (*n* = 2) made no specification. The mean (*M*) age was 42.4 years, with a standard deviation *(SD)* of 11.5 and a range of 21–65 years. Among the total sample, 72.9% (*n* = 255) were nurses or ward managers; 24.6% (*n* = 86) were physicians; and 2.6% (*n* = 9) declared another type of job, such as physiotherapists or pharmacists. The *M* ± *SD* working experience was 14.9 ± 10.5 years, ranging from 0.5 to 40.0 years. More than half of the respondents (53.4%; *n* = 187) reported a full-time employment (employment: 100%); 44.6% (*n* = 156) reported a part-time employment (employment: < 100%); and 2% (*n* = 7) made no specification. The demographic data are shown by country in S2 Table.

### Sound perception

#### Sound levels

**Fig 1:**
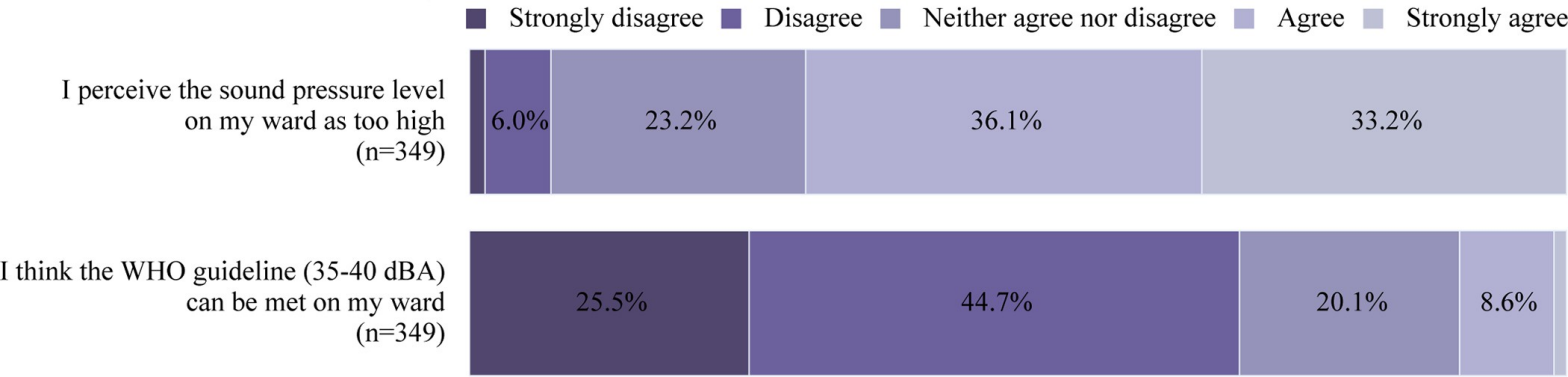
Perception of the sound levels among healthcare professionals. The percentage of healthcare professionals who strongly disagreed, disagreed, neither agreed nor disagreed, agreed, and strongly agreed to the questions asked regarding their perception of the sound levels in their ward is shown. Approximately 1.5% of the healthcare professionals strongly disagreed to the first item, while 1.1% strongly agreed to the second item.

The distribution of the perceptions of the sound level in the ward among the healthcare professionals is illustrated in Fig 1.

The sound levels perceived by the healthcare professionals working in the ICUs varied significantly according to the number of beds in the rooms [chi^2^(2) = 11.367, *p* = 0.003]. The post-hoc analysis revealed a significant difference between the predominantly single- and multi-bed rooms, as the healthcare professionals working in units with single-bed rooms were less likely to rate the sound levels as too high (z = -3.285, *p* = 0.003, r = 0.30) (Table 1).

**Table 1:** Perception of the sound levels according to the hospital characteristics.

Hospital data	% (*n*)	*M* ± *SD*	Statistics^a^	95% CI	Effect size^b^
**Type of unit**					
ICU	74.9% (260)	3.92 (0.99)	F(2) = 0.12, *p* = 0.883	[0.00; 0.01]	r = 0.00
IMC	3.8% (13)	3.92 (0.76)			
No clear separation	21.3% (74)	3.99 (0.94)			
**Specialisation of unit**					
Mixed	68.4% (232)	4.10 (0.87)	F(3) = 1.12, *p* = 0.341	[0.00; 0.03]	r = 0.10
Surgical	15.3% (52)	4.04 (0.95)			
Medical	9.4% (32)	3.94 (0.95)			
Specialised	6.8% (23)	3.65 (1.19)			
**Number of beds**					
≤ 20	79.1% (276)	3.95 (0.98)	t(347) = 0.33, *p* = 0.744	[-0.21; 0.29]	r = 0.02
> 20	20.9% (73)	3.90 (0.90)			
**Room type**					
Mostly single-bed rooms	17.8% (62)	3.58 (1.06)	Chi^2^(2) = 11.37, *p* = 0.003	[0.01; 0.08]	–
Mostly multi-bed rooms	62.8% (219)	4.06 (0.91)			
Equal number of single- and multi-bed rooms	19.5% (68)	3.87 (0.98)			

The perception of the sound levels is grouped on the basis of the hospital characteristics. The data are presented as % (*n*) or means and standard deviations), with the scores ranging from 1 (strongly disagree) to 5 (strongly agree). The percentages were calculated according to the total data set available for each comparison. Abbreviations: intensive care unit (ICU); intermediate care unit (IMC); mean (M); standard deviation (SD); confidence interval (CI).

^a^ Analysis of variances independent-sample t-test, and Kruskal–Wallis rank sum test.

^b^ Pearson’s r.

The healthcare professionals with work experience of > 5 years were more likely to perceive the sound levels as too high than those with ≤ 5 years of experience [*t*(342) = -2.21, *p* = 0.027, r = 0.12] (Table 2).

**Table 2:** Perception of the sound levels according to the demographic characteristics.

Demographic data	*% (n)*	*M* ± *SD*	Statistics^a^	95% CI	Effect size^b^
**Sex**					
Female	66.9% (232)	3.97 (0.94)	t(345) = 1.03, *p* = 0.304	[-1.03; 0.33]	r = 0.06
Male	33.1% (115)	3.86 (1.00)			
**Age**					
≤ 40 years	45.0% (157)	3.84 (0.92)	t(347) = -1.69, *p* = 0.092	[-0.38; 0.03]	r = 0.09
> 40 years	55.0% (192)	4.02 (1.00)			
**Job**					
Nurse	74.7% (254)	3.99 (0.91)	t(338) = -0.79, *p* = 0.429	[-0.32; 0.14]	r = 0.04
Physician	25.3% (86)	3.90 (1.02)			
**Employment**					
Full time (100%)	54.5% (187)	3.88 (0.97)	t(340) = 1.29, *p* = 0.198	[-0.07; 0.34]	r = 0.07
Part time (< 100%)	45.5% (156)	4.01 (0.97)			
**Work experience**					
≤ 5 years	25.3% (87)	3.74 (1.03)	t(342) = -2.21, *p* = 0.027	[-0.50; -0.03]	r = 0.12
> 5 years	74.7% (257)	4.00 (0.94)			

The perception of the sound levels is grouped on the basis of the demographic characteristics. The data are presented as % (*n*), means and standard deviations ranging from 1 (strongly disagree) to 5 (strongly agree). The percentages were calculated according to the total data set available for each comparison. Abbreviations: mean (M); standard deviation (SD); confidence interval (CI).

^a^ Independent-sample t-test.

^b^ Pearson’s r.

When asked in which shift the respondents perceived the sound level as the highest, 89.2% (*n* = 306) responded with the day shift, 7.6% (*n* = 26) with the night shift, and 3.2% (*n* = 11) with the evening shift.

### Disturbance from sound sources

The level of disturbance differed significantly between all sound source groups [Chi^2^(4) = 592.18, *p* < 0.001]. Table 3 shows the post-hoc test results along with the statistical differences between all sound source groups. The short-lasting human sounds were perceived as the most disturbing (*M* = 3.30 ± *SD* 0.81), followed by the devices and their alarms (*M* = 2.67 ± *SD* 0.59), and short-lasting object sounds (*M* = 2.55 ± *SD* 0.68). The largest effect sizes were found between the short-lasting and continuous human sounds (r = 0.83), and between the short-lasting human and continuous object sounds (r = 0.82).

**Table 3:** Disturbance rating of sound sources.

Sound source group^a^	*M* ± *SD*	Statistics^b^	Effect size^c^
**1) Device + alarms**	2.67 (0.59)		
Short-lasting object sounds	2.55 (0.68)	z = -3.62, *p* < 0.001	r = 0.19
Short-lasting human sounds	3.30 (0.81)	z = 11.70, *p* < 0.000	r = 0.63
Continuous object sounds	2.19 (0.56)	z = -12.32, *p* < 0.000	r = 0.66
Continuous human sounds	2.34 (0.49)	z = -9.40, *p* < 0.000	r = 0.50
**2) Short-lasting object sounds**	2.55 (0.68)		
Short-lasting human sounds	3.30 (0.81)	z = 13.30, *p* < 0.000	r = 0.71
Continuous object sounds	2.19 (0.56)	z = -13.05, *p* < 0.000	r = 0.70
Continuous human sounds	2.34 (0.49)	z = -6.44, *p* < 0.001	r = 0.34
**3) Short-lasting human sounds**	3.30 (0.81)		
Continuous object sounds	2.19 (0.56)	z = 15.28, *p* < 0.000	r = 0.82
Continuous human sounds	2.34 (0.49)	z = -15.52, *p* < 0.000	r = 0.83
**4) Continuous object sounds**	2.19 (0.56)		
Continuous human sounds	2.34 (0.49)	z = 5.45, *p* < 0.001	r = 0.29

The disturbance from the different sound sources is categorised into five sound groups: 1) devices and their alarms, 2) short-lasting object sounds, 3) short-lasting human sounds, 4) continuous object sounds, and 5) continuous human sounds. The group values were calculated according to the average value of the individual sound sources (S4 Table). The data are presented as means and standard deviations, with the scores ranging from 1 (not disturbing at all) to 5 (very disturbing). Abbreviations: mean (M); standard deviation (SD).

^a^ Groups 1 to 4, *n* = 349; group 5, *n* = 350.

^b^ Dunn–Bonferroni post-hoc test after a significant Friedman’s test.

^c^ Pearson’s r.

### Sound reduction measures

**Fig 2:**
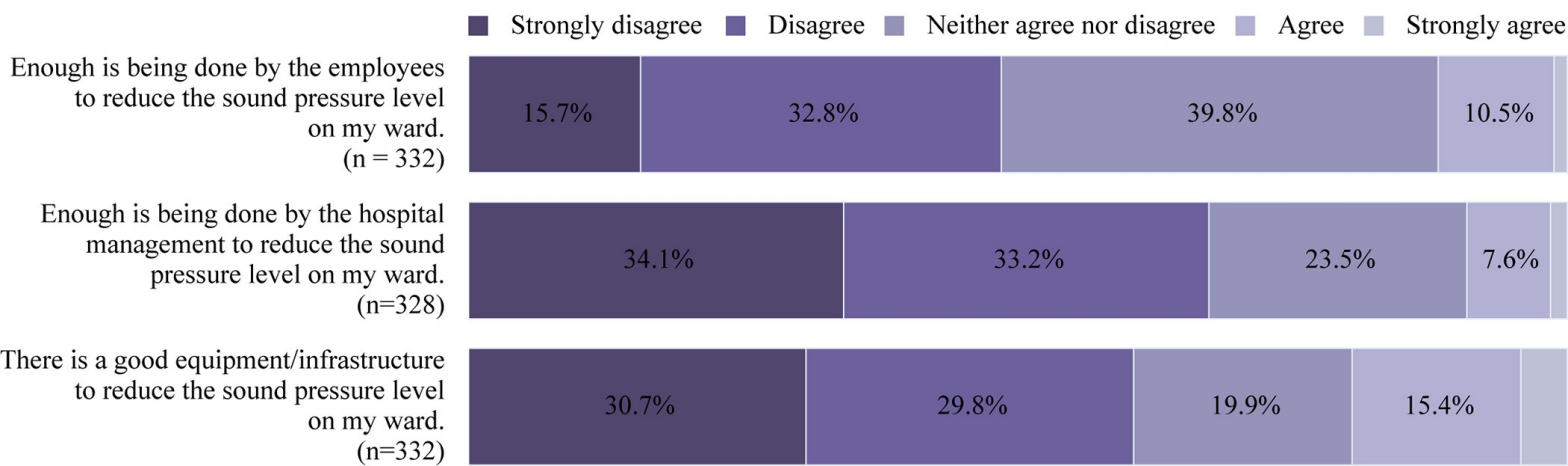
Perception of the sound reduction measures among healthcare professionals. The percentage of healthcare professionals who strongly disagreed, disagreed, neither agreed nor disagreed, agreed, and strongly agreed to the questions asked regarding measures to reduce the sound levels in their ward is shown. Approximately 1.6%, 1.1%, and 4.2% of the of healthcare professionals strongly agreed to the first, second, and third items, respectively.

Fig 2 shows the distribution of the perceptions of the sound-reduction measures in the ward among healthcare professionals.

The potential, feasibility, and the motivation for implementation were significantly greater for reducing medical equipment alarms (*M* = 3.62 ± *SD* = 0.92; *M* = 3.19 ± *SD* = 0.93, and *M* = 3.85 ± *SD* = 0.89, respectively) than for changing staff behaviours and work procedures (*M* = 3.25 ± *SD* = 0.81, *M* = 2.63 ± *SD* = 0.68, and *M* = 3.34 ± *SD* = 0.83) [t(334) = -7.30, *p* < 0.001; t(334) = -11.02, *p* < 0.001; and t(334) = -10.10, *p* < 0.001, respectively) all variables had moderate effect sizes (r = 0.37, r = 0.52, and r = 0.48, respectively) (Table 4).

**Table 4:** Comparison of the sound reduction measures.

	Changing staff behaviours and work procedures^a^	Reducing medical equipment alarms^b^			
Scale	*M* ± *SD*	*M* ± *SD*	Statistics^c^	95% CI	Effect size^d^
Potential	3.25 (0.81)	3.62 (0.92)	t(334) = -7.30, *p* < 0.001	[-0.47; -0.27]	r = 0.37
Feasibility	2.63 (0.68)	3.19 (0.93)	t(334) = -11.02, *p* < 0.001	[-0.66; -0.46]	r = 0.52
Motivation	3.34 (0.83)	3.85 (0.89)	t(334) = -10.10, *p* < 0.001	[-0.60; -0.41]	r = 0.48

The measures were evaluated on the basis of their potential, feasibility, and motivation for implementation. The data are presented, as means and standard deviations, with scores ranging from 1 (not high at all) to 5 (very high). Abbreviations: mean (M); standard deviation (SD); confidence interval (CI).

^a^ *n* = 336.

^b^ *n* = 335.

^c^ Dependent-sample t-test.

^d^ Pearson’s r.

## Discussion

Only few studies have examined the perception of the sound levels in ICUs among healthcare professionals in detail. Herein, our results found that the healthcare professionals were aware of the high sound levels in their work environment and perceived short-lasting human sounds (e.g. calls over long distances or laughs) as the most disturbing, followed by devices and their alarms (e.g. vital sign monitor/alarms or respirator), and short-lasting object sounds (e.g. opening packages or squeaking shoes). Moreover, the healthcare professionals with more work experience and those working in units with mostly multi-bed-rooms were more likely to perceive the sound levels as too high than those with less work experience and those working in units with mostly single-bed rooms. Finally, reducing medical equipment alarms was rated by the healthcare professionals as being the better approach to reduce the sound levels in terms of implementation potential, feasibility, and motivation than changing staff behaviours and work procedures. Taken together, these results clarify how healthcare professionals perceive their work environment and highlight which areas could be targeted to improve their working conditions.

### Sound perception

While there is evidence that healthcare professionals perceive the sound level in ICUs as too high, only few studies have attempted to evaluate what they consider as the most disturbing sound sources and how they perceive them [21, 29, 37]. Kooshanfar et al. [38] determined the sources of noise and the related adverse effects from the perspective of nurses. However, while they asked nurses to rate the major sound sources inside and outside the ward, they did not explore how disturbing the sound sources were. This is an important and relevant distinction highlighting the current gap in the literature. While a sound, such as an alarm, may objectively and subjectively be considered the loudest, that does not imply that it is the most annoying or disturbing source. In this example, a healthcare professional may recognise the necessity of an alarm and thereby not perceive it as annoying; meanwhile, squeaking shoes may be noticeably quieter but may be perceived by the same individual as more frustrating perhaps because the sound provides no task-relevant information.

### Sound levels

Our results regarding the perception of the sound levels agree with previous reports in this field. A study found that 90% of Swiss ICU healthcare professionals perceived sounds as too loud, and 87% thought that sound reduction guidelines were not implemented most of the time [29]. Similarly, a Swedish research group showed that 91% of nurses reported being negatively affected by the noise in their work environment, resulting in irritation, fatigue, concentration problems, tension headache, and burnout [21, 39]. Additional health concerns related to elevated sound levels include tachycardia, hypertension, heart disease, and stroke [18, 19].

In the present study, an explorative approach was used to examine which hospital and demographic characteristics influence the perception of the sound levels among healthcare professionals working in ICUs. It was found that the healthcare professionals working in the ICUs with predominantly single-bed rooms were less likely to perceive the sound levels as too high than those working in the ICUs with predominantly multi-bed rooms. However, the objective measurements of the sound levels did not significantly differ between the healthcare professionals working in units with single-bed rooms and in those with open-bed and two- or four-bed rooms [3, 34]. The role of short-lasting sounds may be responsible for the perceived difference because studies found less occurrences of sounds with a shorter duration in single-bed rooms than in multi-bed rooms [7, 34]. Therefore, healthcare professionals might perceive the environment as less disturbing because fewer coping strategies are needed [17]. The potentially protective effect of working in single-bed rooms among staff needs to be explored in further research.

In addition to the number of beds in a room, work experience was also found to have a significant effect on the perception of the sound levels. The healthcare professionals with more work experience in the ICU were more likely to perceive the sound levels as too high than those with less work experience. In contrast, no difference in age was seen. These results are in line with related reports that showed correlations between noise sensitivity and the number of working years [29]. Therefore, work experience is an influencing factor of noise sensitivity in ICUs, because sound-related stress may accumulate over time [29].

Moreover, 89% of the healthcare professionals indicated that the day shift was the loudest shift in their ward. The difference in the perceived sound level between shifts may be related to the number of sound sources, as seen in single-bed rooms. This could be attributed to the fact that more diverse activities occur in the morning, such as ward rounds, patient admissions and transfers, and nursing care. The objective sound levels are not systematically lower during the night than during the day; this only occurs when fewer patient admissions take place and no urgent interventions are performed [27, 40]. However, further research is needed to explore the effects of the duration and occurrence of noises on shift work.

### Disturbance from sound sources

Understanding how healthcare professionals perceive their work environment is crucial, as they play a central role in reducing the elevated sound levels [3, 4, 40–42]. In the literature, short-lasting and sudden sounds are described as more disturbing for humans than continuous sounds [15, 17]. Additionally, humans perceive sounds with comprehensible content as more disturbing than those without [15]. This supports our findings that the short-lasting human sounds yielded the highest levels of disturbance in the ICU, followed by the devices and their alarms, and short-lasting object sounds. Previous research in ICUs obtained similar results that healthcare professionals perceived telephone conversations, surveillance monitor alarms, and conversations among colleagues as the most disturbing and annoying among 11 common sound sources [29]. Recently, healthcare professionals also stated that the most frequent noises are those from speaking loudly, laughing, and shouting [37]. Similar results were found in studies using observers [34] and those asking patients [8, 9, 43] to identify disturbing sound sources at discharge.

The overall context in which different sound sources occur must also be considered. Herein, the healthcare professionals were asked to elaborate on what exactly disturbed them about the different sound sources (S4 Table). Large numbers of people being present and talking simultaneously during ward rounds, shift changes, and patient admissions and transfers were most frequently mentioned.

### Sound reduction measures

Regarding the current measures to reduce the sound levels in the ICU, around 50% and 70% of the respondents stated that insufficient effort was being made by employees and hospital administrators, respectively. Furthermore, approximately 60% reported that their hospitals did not have good equipment and infrastructure to reduce the sound levels. This shows that healthcare professionals still see a need for improvement at all levels. Specifically, respondents considered the short-lasting human sounds (e.g. laughing or shouting) as the most disturbing sounds during their daily work. This agrees with previous reports that staff behaviour is a major contributor to high sound levels [3, 4, 40, 41]. However, despite the healthcare professionals rating the short-lasting human sounds as the most disturbing sounds, they considered changing staff behaviours and work procedures to have lesser potential, feasibility, and motivation for implementation than reducing medical equipment and alarms. This is crucial particularly because the impact of reducing medical equipment and alarms on the sound levels is unclear, and their role as a major contributor to the overall sound levels is contested [6, 40, 42]. Similarly, the use of patient-lifting devices in healthcare settings was also poorly accepted by nurses despite the known efficacy of these devices [44]. Studies examining this phenomenon identified the lack of acceptance, perceived need, and motivation to use such devices as factors vital to their implementation [45–48].The influence of the managerial level also plays a role in the regulation of the noise levels [46]. Assuming similar circumstances, nurses must first be made aware of the consequences of elevated sound levels and the benefits associated with their reduction to achieve successful outcomes. While various change theories attempt to explain how and why a change is expected to occur, involving individuals in decision-making can decrease resistance [49]. Change agents, or individuals responsible for guiding the change itself, can be very beneficial to ensuring a successful outcome [49].

### Limitations and outlook

A few limitations should be considered regarding the present findings. Owing to voluntary participation in the survey, it cannot be ruled out that healthcare professionals who found the sound levels as particularly disturbing were more likely to participate in the study, adding a nonresponse bias to the results. In the future, the survey should be included within a yearly assessment of the ICU from the perspective of healthcare professionals to avoid such a bias. Nevertheless, the multicentric approach and the large number of participants suggest that high sound levels are indeed a problem in many ICUs. Additionally, the survey was conducted in three German-speaking countries. This does not allow an overall generalisation; nevertheless, statements can be made beyond a national sample, since previous studies on noise in ICUs were conducted in only one country. As a next step, this survey could be translated to additional languages which would allow it to be distributed to numerous countries. Future studies should also gather information pertaining to further distribution of the survey within clinics, so that the response rate may be calculated.

Another limitation is the difficulty of surveying the complex work environment of ICUs, as a large number of sound sources are present simultaneously [4]. Sound sources need to be identified and considered individually to gain a more detailed insight into these factors in future research. Nevertheless, disregarding the overall context or situation in which a sound arises must be avoided. Accordingly, specific work procedures, including patient admissions and daily nursing care, were grouped as one item. However, the exact nature of these sound sources may vary between hospitals or settings, playing a role in how they are perceived. This limits the comparability between responses. Such a trend was also observed with architectural designs and medical devices and their alarms, since these aspects differ between ICUs [33]. Therefore, only general statements about the overall trend could be made regarding these aspects. Future studies should aim to collect additional information regarding procedures and environments in each hospital, so that more context related to sounds can be gathered.

Further research should also conduct multi-method analyses combining objective sound level with subjective sound perceptions reported by healthcare professionals.

## Conclusions

Within ICUs in Germany, Austria, and Switzerland, healthcare professionals, particularly those with more than > 5 years of experience and those working in units with multi-bed rooms, perceive the sound levels in ICUs as too high. Short-lasting human sounds (e.g. laughs or calls over long distances), devices and alarms (e.g. vital sign monitor/alarms or machine sounds), and short-lasting object sounds (e.g. opening and closing doors and pager sounds) are perceived as more disturbing during the daily work of healthcare professionals than continuous human (e.g. conversations between employees or daily nursing care) and continuous object sounds (e.g. cleaning wards or ventilation equipment sounds). While healthcare professionals see a need for reducing the sound levels in their wards, the motivation, feasibility, and potential for implementing behavioural changes to address this problem are low. Healthcare professionals express a desire to reduce medical equipment and alarms to decrease the sound levels.

## Supporting information

S1 Table Hospital data according to country. (PDF)

S2 Table Demographic data according to country. (PDF)

S3 Table Perception of the sound level according to country. Full analysis the perception of sound levels and sound source groups according to country. Between countries there were no significant differences identified.
(PDF)

S4 Table Full list of sound sources. The online survey queried 52 sound sources related to disruption in daily work from the perspective of healthcare professionals in ICUs. Items are listed as they were presented in the survey.
(PDF)
