# Supplementary material for: Perceived sounds and their reported level of disturbance in intensive care units: A multinational survey among healthcare professionals
Source: PLoS One. 2022 Dec 30;17(12):e0279603. doi: 10.1371/journal.pone.0279603 (PMC9803129; doi:10.1371/journal.pone.0279603)
Supplement: S1 Table — (PDF) [file pone.0279603.s001.pdf]

Table S1. Hospital data according to country.

Hospital data[a]	Germany 37.4% (n = 131)	Switzerland 32.0% (n = 112)	Austria 30.6% (n = 107)
**Type of hospital**			
Public[b] or university	80.2% (105)	89.3% (100)	97.2% (104)
Private	17.6% (23)	8.0% (9)	0.9% (1)
Not specified or missing	2.3% (3)	2.7% (3)	1.9% (2)
**Type of unit**			
Intensive care unit (ICU)	66.4% (87)	81.3% (91)	77.6% (83)
Intermediate care (IMC)	–	7.1% (8)	4.7% (5)
No clear separation	33.6% (44)	11.6% (13)	15.9% (17)
Not specified or missing	–	–	1.9% (2)
**Specialisation of unit**			
Mixed	70.2% (92)	75.9% (85)	51.4% (55)
Surgical	13.0% (17)	5.4% (6)	27.1% (29)
Medical	6.9% (9)	8.9% (10)	12.1% (13)
Specialized	7.6% (10)	4.5% (5)	7.5% (8)
Not specified or missing	2.3% (3)	5.4% (6)	1.9% (2)
**Number of beds**			
≤ 20	67.9% (89)	74.1% (83)	98.1% (105)
> 20	32.1% (42)	25.9% (29)	1.9% (2)
**Room type**			
Mostly single-bed rooms	21.4% (28)	16.1% (18)	15.0% (16)
Mostly multi-bed rooms	48.1% (63)	68.8% (77)	73.8% (79)
Equal number of single and multi-bed rooms	30.5% (40)	15.2% (17)	11.2% (12)

Hospital specifications separated for Germany, Switzerland, and Austria. Groups are presented as % (n), n represents the number of participants, and percentages were calculated based on the total sample size for each country.

[a] Not specified or missing data are displayed together, where '–' indicates that no data is available

[b] Public hospitals contain also charitable hospitals
