# Supplementary material for: Perceived sounds and their reported level of disturbance in intensive care units: A multinational survey among healthcare professionals
Source: PLoS One. 2022 Dec 30;17(12):e0279603. doi: 10.1371/journal.pone.0279603 (PMC9803129; doi:10.1371/journal.pone.0279603)
Supplement: S2 Table — (PDF) [file pone.0279603.s002.pdf]

Table S2. Demographic data according to country.

Demographic data[a]	Germany 37.4% (*n* = 131)	Switzerland 32.0% (*n* = 112)	Austria 30.6% (*n* = 107)
**Sex**			
Female	64.9% (85)	67.9% (76)	67.3% (72)
Male	35.1% (46)	32.1% (36)	30.8% (33)
Not specified or missing	–	–	1.9% (2)
**Age**	*M ± SD = 43.35 ± 11.80 Range [21,63]*	*M ± SD = 43.59 ± 10.80 Range [26,65]*	*M ± SD = 39.94 ± 11.53 Range [21,62]*
**Job**			
Nurse	67.2% (88)	69.6% (78)	83.2% (89)
Physician	30.5% (40)	26.8% (30)	15.0% (16)
Other job	2.3% (3)	3.6% (4)	1.9% (2)
**Employment**			
Full-time (100%)	54.2% (71)	48.2% (54)	57.9% (62)
Part-time (< 100%)	45.0% (59)	51.8% (58)	36.5% (39)
Not specified or missing	0.8% (1)	–	5.6% (6)
**Work experience**	*M ± SD = 15.56 ± 10.41 Range [0.5,40.0]*	*M ± SD = 15.19 ± 10.18 Range [0.5,38.0]*	*M ± SD = 13.89 ± 10.92 Range [0.5,40.0]*

Participant demographics separated for German, Swiss, and Austrian healthcare professionals. Groups are presented as % (*n*), whereas *n* represents the number of participants, and percentages were calculated based on the total sample size for each country. Abbreviations: mean (M); standard deviation (SD).

[a] Not specified or missing data are displayed together, '–' indicates that no data is available
