# Supplementary material for: Perceived sounds and their reported level of disturbance in intensive care units: A multinational survey among healthcare professionals
Source: PLoS One. 2022 Dec 30;17(12):e0279603. doi: 10.1371/journal.pone.0279603 (PMC9803129; doi:10.1371/journal.pone.0279603)
Supplement: S3 Table — Full analysis the perception of sound levels and sound source groups according to country. Between countries there were no significant differences identified. (PDF) [file pone.0279603.s003.pdf]

Table S3. Perception of the sound level according to country.

	Germany 37.4% (*n* = 131)	Switzerland 32.0% (*n* = 112)	Austria 30.6% (*n* = 107)			
**Perception of sound**[a]	***M* ± *SD***	***M* ± *SD***	***M* ± *SD***	**Statistics**[b]	**95% CI**	**Effect size**[c]
Perception of sound levels	3.98 (0.95)	3.79 (0.95)	4.04 (1.00)	F(2) = 2.124, *p* = 0.121	[0.00; 0.04]	r = 0.35
1) Devices and their alarms	2.72 (0.61)	2.64 (0.56)	2.63 (0.58)	F(2) = 0.806, *p* = 0.447	[0.00; 0.03]	r = 0.10
2) Short-lasting object sounds	2.45 (0.64)	2.57 (0.63)	2.65 (0.77)	F(2) = 2.521, *p* = 0.082	[0.00; 0.05]	r = 0.38
3) Short-lasting human sounds	3.22 (0.76)	3.41 (0.80)	3.27 (0.88)	F(2) = 1.827, *p* = 0.162	[0.00; 0.04]	r = 0.32
4) Continuous object sounds	2.15 (0.52)	2.14 (0.53)	2.28 (0.63)	F(2) = 2.151, *p* = 0.118	[0.00; 0.40]	r = 0.10
5) Continuous human sounds	2.31 (0.50)	2.40 (0.45)	2.31 (0.51)	F(2) = 1.247, *p* = 0.289	[0.00; 0.03]	r = 0.10

Comparison of sound perception for Switzerland, Germany, and Austria. Groups are presented as *mean* (*SD*=standard deviation) ranging from 1 (not disturbing at all) to 5 (very disturbing). Sound source group values were calculated based on the average of individual sound sources (see S4 Table). Abbreviations: mean (M); standard deviation (SD); confidence interval (CI).

[a] Perception of sound levels and groups 1 to 4, *n* = 349; group 5, *n* = 350

[b] Analysis of variances

[c] Pearson's r
