# Supplementary material for: Perceived sounds and their reported level of disturbance in intensive care units: A multinational survey among healthcare professionals
Source: PLoS One. 2022 Dec 30;17(12):e0279603. doi: 10.1371/journal.pone.0279603 (PMC9803129; doi:10.1371/journal.pone.0279603)
Supplement: S4 Table — The online survey queried 52 sound sources related to disruption in daily work from the perspective of healthcare professionals in ICUs. Items are listed as they were presented in the survey. (PDF) [file pone.0279603.s004.pdf]

Table S4. Full list of sound sources.

Sound sources	% (*n*)	*M* ± *SD*	Rank	Sound source group
**Medical Equipment/Procedures**				
Respirator	96.6% (338)	3.11 (1.04)	7	1
Extracorporeal membrane oxygenation	32.9% (155)	2.65 (1.13)	18	1
Vital signs monitor/alarms	97.7% (342)	3.73 (1.00)	1	1
Dialysis	89.4% (313)	3.24 (1.08)	4	1
Feeding Pump	98.3% (344)	2.47 (1.11)	23	1
Perfusors/Syringe pumps	98.6% (345)	2.99 (1.22)	10	1
Suction devices (e.g., intratracheal, oral)	98.0% (343)	2.59 (1.11)	20	1
Compressed air/ Wall connections	99.1% (347)	2.04 (1.07)	40	1
Drainage/Thoracic drainage	98.6% (345)	1.97 (1.01)	44	1
Vacuum assisted closure-therapy pumps	96.9% (339)	2.19 (1.02)	34	1
Warming blanket	97.1% (340)	2.37 (1.16)	29	1
**Routine procedures**				
Ward round	100% (350)	2.65 (1.06)	17	5
Change of shift reports	99.4% (348)	2.96 (1.04)	11	5
Cardiopulmonary resuscitation	97.7% (342)	2.68 (1.22)	16	5
Intubation	96.9% (339)	2.07 (0.87)	38	5
Complaining/moaning patients	97.4% (341)	3.49 (1.14)	3	3
Patient’s admission/takeover	98.6% (345)	2.42 (0.91)	26	5
Planned patient’s admission	96.3% (337)	1.99 (0.70)	42	5
Patient’s transfer	98.0% (343)	1.98 (0.66)	43	5
External consultation (specialized physician)	97.7% (342)	1.93 (0.71)	46	5
Daily nursing care	98.3% (344)	2.04 (0.76)	41	5
Visits from relatives	97.7% (342)	2.41 (0.94)	27	5
Food distribution	94.6% (331)	1.80 (0.74)	50	4
Cleaning ward	98.9% (346)	2.39 (1.13)	28	4
**Communication**				
Private conversations between employees	98.6% (345)	3.07 (0.94)	8	5

Patient-related conversations between employees	98.3% (344)	2.21 (0.83)	33	5
Communication with patients	96.6% (338)	2.07 (0.85)	39	5
Employee's breaks	97.7% (342)	2.13 (1.03)	36	5
Laughs	97.7% (342)	2.76 (1.10)	15	3
Calls over long distances	97.1% (340)	3.64 (1.13)	2	3
**Ambient sounds**				
Opening packages	99.4% (348)	3.17 (1.18)	6	2
Opening and closing doors	96.6% (338)	2.47 (1.14)	24	2
Opening and closing drawers	98.3% (344)	2.47 (1.05)	22	2
Moving material trolley	97.7% (342)	2.59 (1.01)	19	
Laundry trolleys/laundry boxes	96.9% (339)	2.27 (1.03)	31	4
Operating the bed brake	99.1% (347)	2.12 (1.02)	37	2
Adjusting the bed rail	98.6% (345)	2.24 (1.03)	32	2
Adjusting the bed height	97.4% (341)	1.82 (0.83)	48	4
Faucet/water sounds	96.3% (337)	1.82 (0.82)	49	2
Putting on and taking off protective clothing (including gloves)	98.9% (346)	1.91 (0.89)	47	4
Pushing wheelchair	88.9% (311)	1.80 (0.83)	51	4
Opening and closing curtains	85.4% (299)	1.74 (0.88)	52	2
Change garbage bags	97.4% (341)	2.79 (1.21)	14	4
Shoes (squeaky or loud soles)	99.1% (347)	2.55 (1.08)	21	2
Pager	81.7% (286)	2.82 (1.22)	13	2
Call/bell from patient	96.0% (336)	2.85 (1.10)	12	2
Call/bell visit announcement	96.6% (338)	3.01 (1.17)	9	2
Cell phones not muted	95.1% (333)	3.22 (1.34)	5	2
Coffee machine	94.0% (329)	1.94 (1.03)	45	4
Music/radio/television	96.6% (338)	2.43 (1.13)	25	4
Air conditioning/ventilation equipment	94.9% (332)	2.31 (1.19)	30	4
Sounds from outside (ambulance, helicopter, noisy street)	97.1% (340)	2.16 (1.04)	35	4

List of all 52 sound sources queried in the online survey. The data are presented as % (*n*), where *n* represents the number of participants and percentages were calculated according to $n = 350$. Sound sources are presented as means and standard deviations ranging from 1 (not disturbing at all) to 5 (very disturbing). Rank represents the rating indicating their order of disturbance (where 1 is most disturbing

and 52 is least disturbing). Rank was determined first by the level of the mean and second by the level of the standard deviation. Sound source groups are those used for statistical analyses and presented in S3 Table, namely 1) Devices and their alarms, 2) Short-lasting object sounds, 3) Short-lasting human sounds 4) Continuous object sounds, 5) Continuous human sounds). Abbreviations: mean (M); standard deviation (SD).
